# A simplified cervix model in response to induction balloon in pre-labour

**DOI:** 10.1186/1742-4682-10-58

**Published:** 2013-09-26

**Authors:** James Andrew Smith

**Affiliations:** 1 Department of Electrical & Computer Engineering, Ryerson University, Toronto, Canada

**Keywords:** Balloon dilator, Cervix, Pre-labour, Latent phase of labour, Labour induction, Dilation, Effacement

## Abstract

**Background:**

Induction of labour is poorly understood even though it is performed in 20% of births in the United States. One method of induction, the balloon dilator applied with traction to the interior os of the cervix, engages a softening process, permitting dilation and effacement to proceed until the beginning of active labour. The purpose of this work is to develop a simple model capable of reproducing the dilation and effacement effect in the presence of a balloon.

**Methods:**

The cervix, anchored by the uterus and the endopelvic fascia was modelled in pre-labour. The spring-loaded, double sliding-joint, double pin-joint mechanism model was developed with a Modelica-compatible system, MapleSoft MapleSim 6.1, with a stiff Rosenbrock solver and 1E-4 absolute and relative tolerances. Total simulation time for pre-labour was seven hours and simulations ended at 4.50 cm dilation diameter and 2.25 cm effacement.

**Results:**

Three spring configurations were tested: one pin joint, one sliding joint and combined pin-joint-sliding-joint. Feedback, based on dilation speed modulated the spring values, permitting controlled dilation. Dilation diameter speed was maintained at 0.692 cm·hr^-1^ over the majority of the simulation time. In the sliding-joint-only mode the maximum spring constant value was 23800 N·m^-1^. In pin-joint-only the maximum spring constant value was 0.41 N·m·rad^-1^. With a sliding-joint-pin-joint pair the maximum spring constants are 2000 N·m^-1^ and 0.41 N·m·rad^-1^, respectively.

**Conclusions:**

The model, a simplified one-quarter version of the cervix, is capable of maintaining near-constant dilation rates, similar to published clinical observations for pre-labour. Lowest spring constant values are achieved when two springs are used, but nearly identical tracking of dilation speed can be achieved with only a pin joint spring. Initial and final values for effacement and dilation also match published clinical observations. These results provide a framework for development of electro-mechanical phantoms for induction training, as well as dilator testing and development.

## Background

Even though labour is induced in over 20% of women in the United States [1] both general labour dynamics and the more specific dynamics of the cervix are poorly understood [2]. Even under controlled conditions with static models, practitioners have poor accuracy in identifying dilation levels and compliance [3].

To promote systematic exploration and analysis of cervical dynamics a new model is proposed here, expressed in explicit analytic form, which strikes a balance between the intuitive qualitative descriptions typically used by clinicians [2], and numerically intensive finite-element models [4]. This lays the foundation for a framework for modelling in simulation and with electromechanical phantoms.

In this study we will concentrate on pre-labour ([5], p 185–186) (or the *latent* stage of labour) and the application of balloon dilators during this phase to induce active labour. Labour induction techniques are varied, with balloons being one of the oldest contemporary techniques [6]. The median duration *can* last from about 5 hours for a woman who has previously given birth to about 8 hours for a woman giving birth for the first time ([5], p. 186). The transition from pre-labour to active labour is typically assumed to have occurred when the cervical diameter is anywhere from 3 to 5 cm [5,7].

It is generally admitted that while cervical dilation is a convenient measurand for tracking progress of labour, it is insufficient. Head-to-cervix force, uterine activity, effacement rates, and dilation rates all appear to play a role in birth mode or outcome [8]. The exact and distinct nature of these measurands is unclear, leading to a wide range of labour induction intervention methods [9] and on-going efforts at comparison [10-12]. One of these methods, the indirect balloon dilator, holds advantages over other methods in that it appears to mimic processes for cervical dilation related to head-to-cervix forces. These processes tend to be slower than those seen in pharmacological approaches or direct radial dilators, but are potentially safer [13] if care is taken to minimize risk of infection inherent with insertion of foreign objects in the endocervical canal [9].

### Balloon dilators

**Figure 1:**
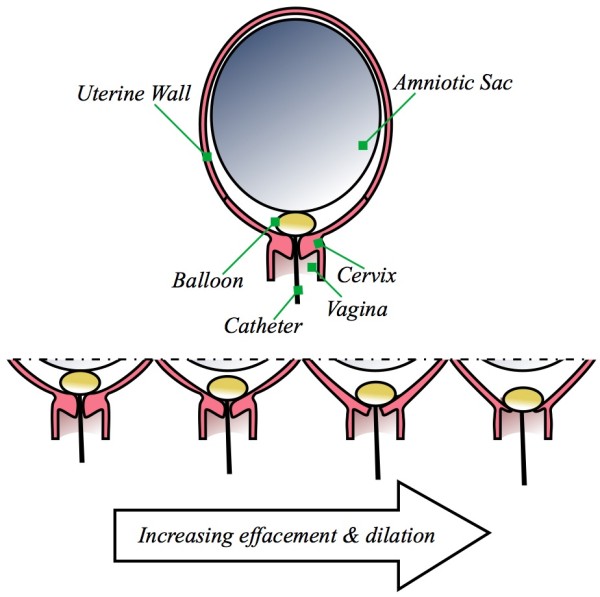
**Relevant anatomy and balloon dilator applied to the cervix.** A balloon catheter is placed in the space between the amniotic sac and the interior os of the cervix to induce labour. From left to right, as the cervix softens it effaces (thins) and dilates (opens), permitting the balloon to descend and eventually exit.

The contemporary Foley catheter balloon dilator originates from designs first introduced in the 1850s [14,15]. There are two major methods for dilation: direct and indirect. Direct involves placing a device within the endocervical canal and expands the canal through a laterally-applied force [16], but is typically no longer practiced in labour induction because of the dangers it presents. The safer indirect, or "from above" [15], method involves a balloon placed in the extra-amniotic space above the interior os of the cervix, as shown in Figure 1. Its presence engages an internal reaction in the cervix, similar to that seen during normal pre-labour allowing the cervix to efface (thin) and dilate (open). It is possible for force to be applied by the balloon to the interior os even without explicit external traction, due to the balloon filling the extra-amniotic space and transferring force from the amniotic sac to the interior os. The indirect, or from above, method is examined here.

#### ***Tractive force: amount and duration***

The amount of explicit external tractive force varies, from none [17], to "minimal" [18], to about 0.5 kg (approx. 5 N) [19] or more [20], but is often not directly specified [21]. The tractive force most often appears to be achieved by taping the end of the lumen to the patient’s thigh, leaving few practical methods for systematic measuring of force measurement. Typically, one waits many hours [22] for the balloon to be expelled naturally: from half-an-hour [23] to ten hours [24], with suggestions of six hours being an acceptable upper limit before attempting other forms of intervention [25]. Expulsion of the balloon occurs when dilation reaches approximately four centimetres. This is comparable to the non-induced pre-labour phase of labour [26].

In this study we simulate a 0.5 kg balloon-applied tractive force, directed to the inner os of the cervix, leading to the indirect dilation of the cervix through a softening of the tissue. Final dilation diameter (4.5 cm) and effacement (2.25 cm) and time scale (about 7 hours) are similar to what is seen in practice. The details of the model are presented below.

## Main text

Laplace’s law, for vessels under pressure, has been suggested as a modelling framework [13,27]. In the same vein, more numerically intensive finite-element models have recently been produced [4]. Both of these approaches, however, did not examine the use of balloon dilators during pre-labour.

Cervical anatomy and dynamics are highly variable, with some correlation to gestational age and parity [2]. The proposed model’s parameters can easily be adapted to other configurations and dynamic characteristics given the combination of schematic and analytic expressions. In the context of this manuscript we make the following assumptions. The subject is assumed to be at full term of her pregnancy, that is, 39 to 40 weeks. Rovas *et al.* showed that cervical width is relatively constant from weeks 31 through 41 and that is similar in women who have given birth before and those who have not. Cervical width is therefore assumed to be 4.5 cm [28]. As less than 40% of women in Rovas *et al.*’s study had an open cervix at 39–40 weeks, we will assume that the endocervical canal is initially closed and no funnelling is present. Therefore, cervical wall thickness is 2.25 cm. Here, we assume that cervical length, at 39 to 40 weeks, is approximately half-way between that for women who have previously given birth (1.2 cm) and women giving birth for the first time (3 cm) [28]. For convenience, we will assume that length equals wall thickness: 2.25 cm.

Cervical mass had to be estimated since no published data on cervical mass at term were found after a literature search and consultations with a number of health professionals. To estimate cervical mass, we assume that mass distribution is uniform throughout the uterus, including the cervix. The uterus, without its contents, is assumed to have a mass of 1 kg and to be a 30 cm by 23 cm by 20 cm ellipsoid [29]. The uterine wall is assumed to be 0.36 cm thick [30]. Assuming a cervix in the shape of a cylinder 4.5 cm wide by 2.25 cm high, the at term cervix will have a mass of 0.027 kg.

While the effect of cervix mass on dynamics with a timescale measured in hours is small, it is important to note that this model is also meant for application to electro-mechanical models in which the timescale can be greatly accelerated. For instance, in a current prototype, full dilation can be achieved in less than a minute. For training and device testing purposes it is important that the model be applicable at both short and long timescales, hence the inclusion of the cervix mass.

Given the restriction of the model to pre-labour, it is assumed that dilation will not exceed Rovas *et al.*’s 4.5 cm cervix width, at which point the balloon will exit. Therefore, the cervix is assumed to be anchored to the lower uterus and the endopelvic fascia in such a manner that the anchor point does not move. This permits the model to exclude the dynamic effects associated with movement of the anchor. This assumption stops being valid as the "active" phase of labour begins. Neither friction nor surface deformations are modelled in this manuscript, leaving such details to future work.

### 

#### ***Simplification of the cervix through symmetry: modelling one quarter of the cervix***

**Figure 2:**
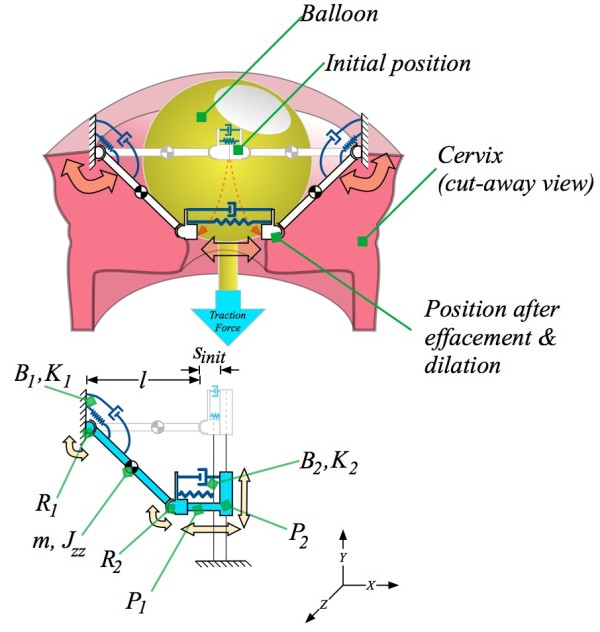
**Simplification from a "half-model" cervix to a "quarter-model" cervix.** Above is the "half-model" superimposed on a balloon and cervix. The half-model approximates the dilation and effacement in reaction to the balloon as a series of linkages, pin joints, sliding joints, springs and dampers. Below is the further-reduced "quarter-model", which captures the same effects but with fewer components. The mathematical model described in this paper uses the "quarter model". It has one quarter the cervix mass, one quarter the compliance, and bears one quarter the traction force.

The modelling objective is to reproduce the behaviour seen during pre-labour in which a balloon has been introduced to soften the cervix, with as few degrees of freedom as possible. As the cervix becomes more compliant, the cervix dilates to a level which permits the balloon to exit the cervix. The behaviour of the cervix can be approximated in a plane by a pair of compliant multi-joint arms that move in response to a tractive force from above. This is illustrated in the upper portion of Figure 2. One can then assume that another pair of arms, in a perpendicular plane behave similarly, with each arm responsible for the reaction to one quarter of the tractive force from the balloon. In the bottom portion of Figure 2 the model is simplified to reflect the one-quarter support perspective. This simplifies the mathematical model to a double-sliding-joint, double-pin-joint, spring-loaded system, discussed in the following section.

The "quarter model" presented here the contains the minimum number of degrees of freedom which permit dilation in a direction perpendicular to the tractive loading typical of Foley-type balloon dilators. As can be seen in the bottom portion of Figure 2, one pin joint and one sliding joint contain spring-damper pairs. One controls pivoting at the point where the cervix is anchored to the remainder of the uterus, as well as to the endopelvic fascia. The other, across a sliding-joint, approximates the compliance that spans laterally, across the endocervical canal.

#### ***The mathematical model***

Assuming symmetric geometry and dynamics, the model can be simplified to a single, stretchable arm. The model is composed of two pin joints and two sliding joints. This is illustrated schematically in Figure 2.

The dynamics are expressed as a set of differential algebraic equations with constraint reactions, expressed in general, high-level form as:

(1) $$M\cdot \frac{dp}{dt}+C^{T}\cdot f=F$$

where *M* is the mass matrix, $\frac{dp}{dt}$ is the time derivative of generalized speeds, *C*^*T*^ is the transposed matrix of constraint reactions, *f* are the reaction forces, and *f* contains the external loading forces.^a^ The system is described in Eq. 1 by four generalized coordinates, *Q*, which are, in turn, coupled by the three algebraic constraints of Eq. 4, yielding a single degree of freedom. The generalized coordinates, *Q* are defined as

(2) $$Q=\left[\begin{array}{c} s_{P_{1}}(t)\\ s_{P_{2}}(t)\\ \theta_{R_{1}}(t)\\ \theta_{R_{2}}(t) \end{array}\right]$$

where $\theta_{R_{1}}(t)$ and $\theta_{R_{2}}(t)$ are the angles of the first and second pin joints, respectively. The length of the two sliding joints are defined as $s_{P_{1}}(t)$ and $s_{P_{2}}(t)$. The time derivative of the generalized speeds, $\frac{dp}{dt}$, is the second time derivative of *Q*:

(3) $$\frac{dp}{dt}=\left[\begin{array}{c} \frac{d^{2}}{dt^{2}}s_{P_{1}}(t)\\ \frac{d^{2}}{dt^{2}}s_{P_{2}}(t)\\ \frac{d^{2}}{dt^{2}}\theta_{R_{1}}(t)\\ \frac{d^{2}}{dt^{2}}\theta_{R_{2}}(t) \end{array}\right]=\left[\begin{array}{c} {\ddot{s}}_{P_{1}}(t)\\ {\ddot{s}}_{P_{2}}(t)\\ {\ddot{\theta }}_{R_{1}}(t)\\ {\ddot{\theta }}_{R_{2}}(t) \end{array}\right].$$

The three position, or kinematic, constraint equations are described as follows:

(4) $$\;\left[\;\begin{array}{c} \text{cos}(\theta_{R_{1}}(t))\;\cdot \;s_{\text{init}}+\text{cos}(\theta_{R_{1}}(t))\;\cdot \;l-l+\text{sin}(\theta_{R_{1}}(t))\cdot s_{P_{2}}(t)-s_{P_{1}}(t)\cdot \text{cos}(\theta_{R_{2}}(t))]\\ -\text{sin}(\theta_{R1}(t))\;\cdot \;s_{\text{init}}-\text{sin}(\theta_{R_{1}}(t))\;\cdot \;l-s_{P_{1}}(t)\cdot \text{sin}(\theta_{R_{2}}(t))+\text{cos}(\theta_{R_{1}}(t))\cdot s_{P_{2}}(t)\\ \text{sin}(\theta_{R_{1}}(t))\;\cdot \;\text{cos}(\theta_{R_{2}}(t))+\text{cos}(\theta_{R_{1}}(t))\;\cdot \;\text{sin}(\theta_{R_{2}}(t)) \end{array}\;\right]\;=\;\left[\begin{array}{c} 0\\ 0\\ 0 \end{array}\right]$$

where *s*_init_ is the initial radius of the opening of the cervix, *P*_2_. We assume that *s*_init_ is zero in the simulations which follow. The length, *l*, is the initial thickness of the cervical wall.

The mass matrix, *M*, contains both mass and moment of inertia, is defined as:

(5) $$M=\left[\begin{array}{cccc} 0 & 0 & 0 & 0\\ 0 & 0 & 0 & 0\\ 0 & 0 & J_{\text{zz}}+\frac{1}{2}\cdot {\left(\frac{l}{2}\right)}^{2}\cdot m & 0\\ 0 & 0 & 0 & 0 \end{array}\right]$$

where *l* is the length of the link between the two pin joints, with a mass located in the geometric centre. The moment of inertia about the *z*-axis is calculated as a thin cylinder, $J_{\text{zz}}=\frac{1}{12}\cdot m\cdot l^{2}$. The mass, *m*, is one quarter of the total cervix mass.

The external loads are described in the vector, *F*, from the right hand side of Eq. 1, as

(6) $$F=\left[\begin{array}{c} K_{2}\cdot s_{\text{init}}-K_{2}\cdot s_{P_{1}}(t)-B_{2}\cdot \frac{d}{d^{t}}s_{P_{1}}(t)\\ F_{\text{Traction}}\\ -\frac{1}{2}\cdot \text{cos}(\theta_{R_{1}}(t))\cdot \frac{l}{2}\cdot g\cdot m-K_{1}\cdot \theta_{R_{1}}(t)-B_{1}\cdot \frac{d}{d^{t}}\theta_{R_{1}}(t)\\ 0 \end{array}\right]$$

where the first row describes the rectilinear force that develops across the endocervical canal along *P*_2_, due to the stretching of the spring-damper, *K*_2_ and *B*_2_. The traction force in the second row is assumed to be wholly due to the constant pulling action by a 0.5 kg mass along the gravity vector, supported by four equal sections of the cervix. Therefore, *F*_Traction_ = 0.25·0.5·9.81 = 1.23 N. The third row describes the moment about the first pin joint, anchored by the uterus and endopelvic fascia, consisting of an angle-dependent mass term and the spring-damper stretching moment about *R*_1_ due to *K*_1_ and *B*_1_.

The four-by-three-element constraint matrix, *C*^*T*^, is defined as

(7) $$C^{T}=\left[\begin{array}{ccc} \text{cos}(\theta_{R_{2}}(t)) & \text{sin}(\theta_{R_{2}}(t)) & 0\\ -\text{sin}(\theta_{R_{1}}(t)) & -\text{cos}(\theta_{R_{1}}(t)) & 0\\ -s_{P_{1}}(t)\cdot \text{sin}(\theta_{R_{2}}(t)) & l+s_{P_{1}}(t)\cdot \text{cos}(\theta_{R_{2}}(t)) & 1\\ -s_{P_{1}}(t)\cdot \text{sin}(\theta_{R_{2}}(t)) & s_{P_{1}}(t)\cdot \text{cos}(\theta_{R_{2}}(t)) & 1 \end{array}\right]$$

Finally, the reaction force vector is

(8) $$f=\left[\begin{array}{c} {\text{Fx}}_{R_{2}}(t)\\ {\text{Fy}}_{R_{2}}(t)\\ {\text{Mz}}_{P_{1}}(t) \end{array}\right]$$

where ${\text{Fx}}_{R_{2}}(t)$ and ${\text{Fy}}_{R_{2}}(t)$ are the *x*-axis and *y*-axis reaction forces at the second pin joint, *R*_2_, and ${\text{Mz}}_{P_{1}}(t)$ is the reaction moment at the first sliding joint, *P*_1_.

The model parameters are found in Table 1. In the following section the model is simulated.

**Table 1:** Simulation parameters for the quarter model

**Parameter**	**Value**	**Units**
Cervical wall thickness	2.25	cm
Initial endocervical canal opening	0	cm
Mass	0.07	kg
Moment of inertia	2.9E-7	kg·m^2^
Pin joint damping	10	N·m·s·rad^-1^
Sliding joint damping	10	N·s·m^-1^
Gravity	-9.81	m·s^-2^
Traction	1.23	N

### Results & analysis

Feedback control has been implemented to control dilation rate throughout the pre-labour phase. Using the model outlined above three cases have been examined: feedback control through only a sliding joint spring, feedback control through only a pin joint spring and, finally, feedback control through both pin joint and sliding joint springs.

**Figure 3:**
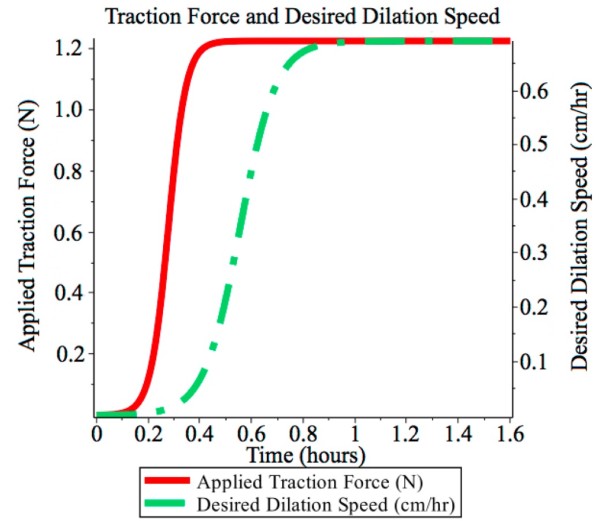
**Ramping of traction force.** Applied traction force and desired dilation speed. Both smoothly ramp up during the initial stages of pre-labour.

Dilation rate is controlled at 0.692 cm · hr^-1^ over approximately 6.5 hours. An additional half-hour is added to permit gradual ramping-up of the uterine force and dilation rate, shown in Figure 3, leading to a total simulation time of 7 hours.

#### ***Effect of varying compliance values on cervical dilation and effacement***

The model assumes that the two main spring constants will increase in compliance (decrease in stiffness) over time, in response to the presence of the balloon. As the compliance increases the traction applied to the balloon acts to deform the cervix, creating both dilation and effacement effects. The balloon descends during the combination of effacement and dilation, continuing to apply the traction force until the end pre-labour when effacement and dilation are complete. A continuum of possible compliance trajectories over time is possible. In the next section simulations are conducted on three scenarios, permitting dilation and effacement to be controlled through the changing of the two spring constants.

#### ***Time-varying compliance trials***

Published cervicograms typically show a constant dilation rate during pre-labour [26]. Sometimes the dilation rate is specified numerically (e.g. Peisner and Rosen specify between 1.2 and 1.5 cm·hr^-1^. [7]). Here, we examine a pre-labour duration of 7 hours (6.5 hours plus a half-an-hour to allow for ramping up of applied traction force and desired dilation to steady-state), resulting in a dilation diameter rate of 0.692 cm·hr^-1^. To avoid large transients in the response of the cervix model two ramping functions, shown in Figure 3, were introduced. The gradual application of the balloon’s traction force is mimicked through a smooth ramping function. The response of the cervix, in the form of the desired dilation velocity also smoothly transitions to the steady state value of 0.692 cm·hr^-1^.

Closed-loop feedback control is used to ensure that dilation rate is near-constant for the majority of the induction. Three scenarios were examined: (1) a controlled pin-joint spring with no sliding spring, (2) a controlled sliding spring with no pin-joint spring, and (3) both controlled pin-joint and controlled sliding springs. Simulations were conducted with MapleSim 6.1’s numeric solver set to Rosenbrock (stiff) with 1E-4 absolute and relative tolerances.

**Figure 4:**
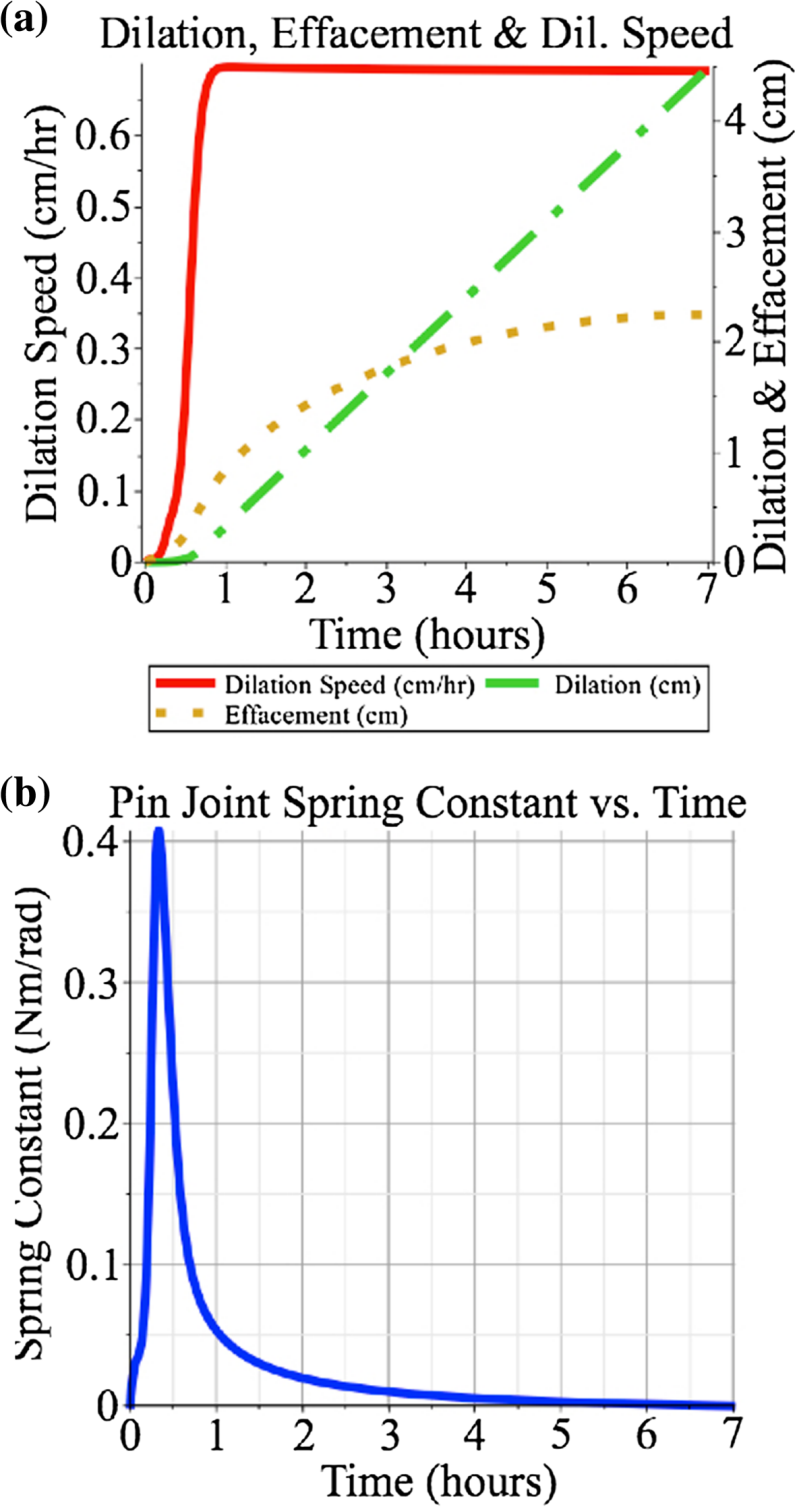
**Feedback-based control of the pin joint spring, with no sliding joint spring.** Feedback-based control of only the pin joint spring, with dilation speed ramping up to a steady-state of 0.692 *cm*·*hr*^-1^ shown in **(a)**. The maximum spring constant, shown in **(b)**, is 0.41 N·m·rad^-1^.

In the controlled pin joint spring case the dilation diameter rate^b^ was set to 0.692 cm·hr^-1^. The dilation rate is measured within the model, error is calculated with respect to a desired rate. A feedback gain of 20 N·s·rad^-1^ is used, converting the feedback error into a spring constant value in N·m·rad^-1^. This gain value was chosen because it was found that lower values led to poor tracking of the desired dilation velocity as it ramped up initially and higher values did not significantly improve tracking error. As is shown in Figure 4, the dilation rate follows the desired values, both during ramping up and during steady-state, and the dilation and effacement are 4.5 cm and 2.25 cm respectively, at the end of the simulation. The maximum spring constant value was 0.41 N·m·rad^-1^.

**Figure 5:**
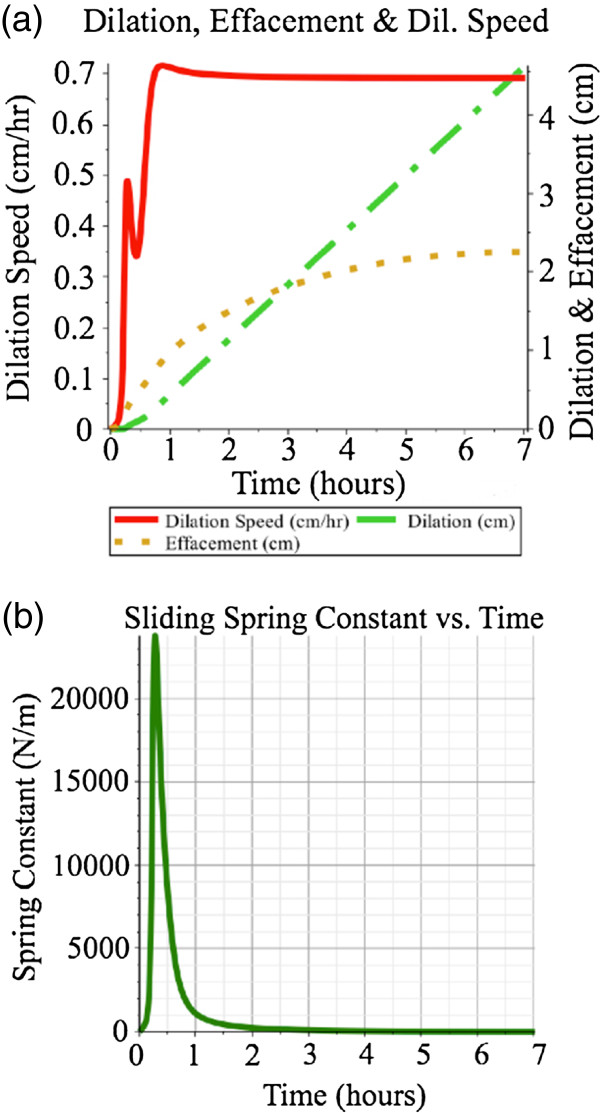
**Feedback-based control of the sliding joint spring, with no pin joint spring.** Feedback-based control of only the sliding joint spring, with dilation speed ramping up to a steady-state of 0.692 *cm*·*hr*^-1^, shown in **(a)**. The maximum spring constant, shown in **(b)**, is 23800 N·m^-1^.

In the controlled sliding spring case the dilation rate is controlled in a similar fashion to the pin-joint spring case. The dilation rate is measured, error determined between it and the desired dilation diameter rate (0.692 cm·hr^-1^) and passed through a proportional feedback gain of 100000 N·s·m^-2^ to yield a varying spring constant value in *N*·m^-1^. Smaller gains produce spikes in the initial dilation speed, while larger gains did not show improved tracking performance. As is shown in Figure 5, the dilation rate shows good tracking after the initial ramping function, and the dilation and effacement are 4.5 cm and 2.25 cm, respectively, at the end of the simulation. The maximum spring constant value was 23800 N·m^-1^.

**Figure 6:**
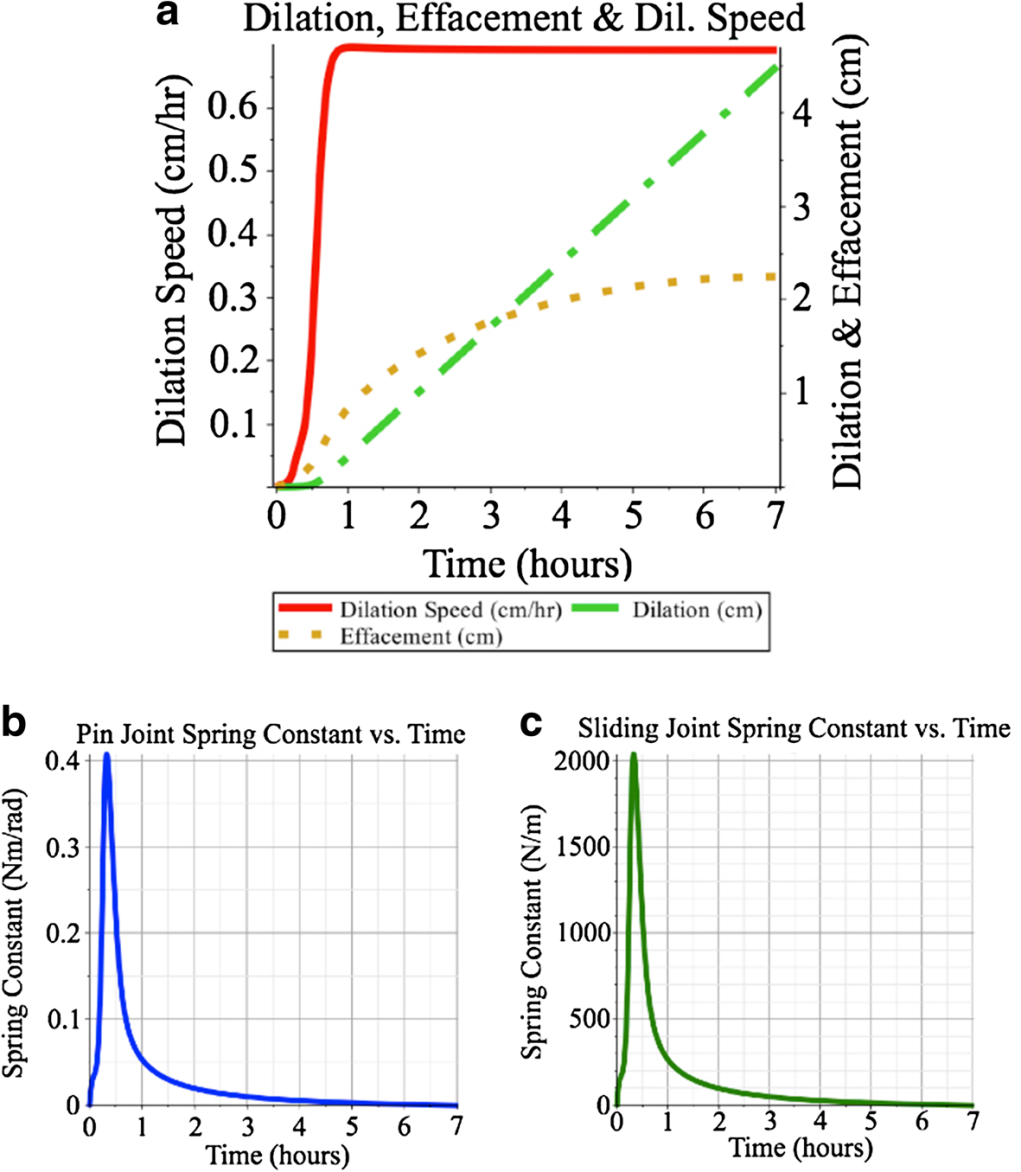
**Feedback-based control of the both sliding spring and pin joint spring.** Feedback-based control of both pin joint and sliding joint springs, with dilation speed ramping up to a steady-state of 0.692 *cm*·*hr*^-1^, shown in **(a)**. Spring constants peak early to counter the more significant torque produced by the traction force. Maximum spring constants are 0.41 N·m·rad^-1^ and 2000 N·m^-1^ and for the pin joint **(b)** and sliding joint **(c)** springs, respectively.

Thirdly, the case in which both springs were controlled with feedback based on dilation rate was examined. The two feedback paths described above were applied in parallel, using the gains specified above. As is shown in Figure 6, the dilation rate follows both the ramping-up values and the dilation and effacement are 4.5 cm and 2.25 cm, respectively, at the end of the simulation. Using the feedback gains specified above, the maximum spring constants are 2000 N·m^-1^ and 0.41 N·m·rad^-1^, respectively. Of course, a continuum of gains could be used and these could be time-varying. For instance, if the pin joint spring gain were held at 20 N·s·rad^-1^, the sliding joint spring gain could be reduced from 100000 N·s·m^-2^, resulting in higher spring constants for the pin joint but lower spring constants in the sliding joint. Conversely, the sliding joint’s feedback gain could be held constant at 100000 N·s·m^-2^ and the pin joint’s feedback gain could be reduced. Performance in this latter case would be good during steady-state but would exhibit moderate error in dilation speed during the ramping-up phase, as shown in Figure 5.

At the beginning of the labour process the torque produced by the traction force on pin joint *R*_1_ is very large because it is completely perpendicular to the moment arm. Either spring in this model can counter the traction force while maintaining a low error in the desired dilation speed. It takes a much smaller feedback gain on the pin joint’s spring to produce the necessary counter-torque necessary to have the cervix hold the balloon in place. A larger effort, in the form of a much larger feedback gain is required by the spring in the sliding joint to produce the same effect. This corresponds to the highest spring constant values, *K*_1_ and *K*_2_, shown in Figures 6b and 6c. In addition, when comparing the three scenarios, the lowest spring constant values are achieved when two springs are used, but nearly identical tracking of dilation speed can be achieved with only a pin joint spring. Worst performance is found when only the sliding joint spring is used.

#### ***Comparison with clinical data***

There is little quantifiable clinical data that describes the dynamics of the interaction between balloon dilator and cervix. What quantified data is available has been used to develop this model. The amount of explicit external tractive force in the model has been set to 0.5 kg (approx. 5 N) [19] the most commonly cited quantified value. The typical time taken for a balloon to be expelled varies from half-an-hour [23] to ten hours [24], while the typically cited range for pre-labour without intervention is 5 to 8 hours. Therefore, 6.5 hours, a value in the mid portion of both ranges, was chosen for simulation.

Because cervicograms typically show dilation progressing at a constant rate it was important that the simulations result in constant dilation rates, as well. While Peisner and Rosen found rates of between 1.2 and 1.5 cm·hr^-1^[7] the rate used in this simulation (in order to ensure constant dilation throughout the duration of pre-labour) had to be set to 0.692 cm·hr^-1^. Had the Peisner and Rosen values been used with this model and had it been assumed that the dilation rate was constant throughout pre-labour, the simulated pre-labour would have lasted under four hours.

When comparing the effacement curves in Figures 4, 5 and 6 to the dilation curves one can see that the effacement effect is more noticeable earlier than the dilation effect. This corresponds to clinical observations that "cervical effacement precedes significant dilation" [31].

The simulation ends when the cervix model dilates to 4.5 cm, which is within the acceptable range for transition from pre-labour to active labour (e.g. 3 cm to 5 cm). The 100% effacement value used in simulation agrees with the upper bounds given in [32].

While this model permits any arbitrary trajectory of compliance values versus time to be programmed, it does not help to answer *why* the cervix responds to the presence of the balloon. It is the author’s perspective that answering this question is key to understanding when intervention should or should not be performed. This will be the objective of future work.

At the moment, there is no other mathematical model which examines the descent of an induction balloon as the cervix softens during pre-labour. The closest models are those by Gee [13,27], using Laplace’s law for a spherical pressure vessel, and finite element analysis approach House *et al.*[4]. Both of these examine the reaction of the cervix to pressure exerted by the uterus, and neither is in the context of induction of labour, nor during pre-labour, nor with respect to balloon dilation.

Unlike the models proposed by Gee or House, the structure of this model lends itself well to the inclusion of variable compliance mechatronic devices [33] in the development of cervix phantoms. These phantoms could potentially be used to explore the dynamics of the cervix during pre-labour, with or without intervention.

## Future work towards a physical training simulator

As discussed earlier, work is proceeding on physical prototypes capable of controlled dilation and effacement. These results provide a framework for further development of electro-mechanical phantoms for physical training or dilator testing. It is envisioned that a three-dimensional physical prototype can be devised using four quarter models wrapped in a compliant covering. Small motors at the pin and sliding joints can be programmed to mimic springs and dampers through a proportional-derivative algorithm. This physical model will also be useful in the development of contact models, including characterization of friction between the balloon and the cervix.

## Conclusions

A model has been proposed and evaluated to represent cervical dilation in response to the presence of a balloon dilator during the pre-labour stage of labour. Simulations have been run to mimic a 6.5 hour pre-labour phase (with an additional half-an-hour for initialization) in response to a small, fixed external balloon-applied traction force. The model contains feedback pathways which permit the dilation rate to be controlled in response to the presence of constant traction applied to the interior os via the balloon. Dilation diameter of 4.5 cm and effacement of 2.25 cm were achieved with a 0.692 cm·hr^-1^ dilation rate. It was shown that compliance could be controlled at either the pin joint located at the junction between the uterus, the endopelvic fascia and the cervix, or along the sliding joint that spans laterally across the endocervical canal. When comparing the three scenarios, the lowest spring constant values are achieved when two springs are used, but nearly identical tracking of dilation speed can be achieved with only a pin joint spring. Worst performance is found when only the sliding joint spring is used. These results can be applied to the development of new electromechanical cervix phantoms for the study of cervical dynamics during pre-labour.

## Endnotes

^a^ Note that the · operator is an explicit multiplication and not the dot product.

^b^ Note that while the dilation rate is given for cervix diameter, in order to be consistent with existing clinical data, the *radius* dilation rate was controlled in the model. The radius dilation rate value is half the diameter dilation rate.

## Competing interests

The author declares that he has no competing interests.

## Authors’ information

James Andrew Smith is an Assistant Professor in Electrical and Computer Engineering at Ryerson University. He is also the stream coordinator for Ryerson’s newly accredited biomedical engineering program. His PhD is in Mechanical Engineering from McGill University and he undertook a postdoctoral fellowship in Sports Science in Jena, Germany. His research interest is in the development of electromechanical surrogates / phantoms for research in biomechanics and obstetrics.
